# An Integrated DAIDS Laboratory Oversight Framework: Application of the DAIDS GCLP Guidelines

**DOI:** 10.1089/aid.2024.0041

**Published:** 2024-11-06

**Authors:** Naana Cleland, Nina Kunwar, Usha Sharma, Jamal Dejli, Milton Maciel, Daniella Livnat, Judith Miller, Keith Crawford, Fatima Jones, M. Patricia D’Souza

**Affiliations:** Division of AIDS, National Institute of Allergy and Infectious Diseases (NIAID), Bethesda, Maryland, USA.

**Keywords:** Clinical Trials, DAIDS GCLP guidelines, GCLP training, GCLP audit, laboratory performance, quality assurance

## Abstract

The Division of AIDS (DAIDS) Good Clinical Laboratory Practice (GCLP) Guidelines establish a framework to guide the oversight of laboratories supporting DAIDS-sponsored clinical research or trials. Compliance with these guidelines promotes data reliability, consistency, and validity, and the safety of the clinical research or trial participants and laboratory staff, as well as ensures adherence to regulatory requirements. This article describes the application of the DAIDS GCLP Guidelines, the DAIDS Integrated Laboratory Oversight Framework, and the coordinated efforts of the collaborative oversight team of laboratory experts to support and monitor the performance of over 175 participating laboratories worldwide.

Data from two self-administered online surveys conducted in 2017 and 2023 assessed the laboratory staff’s experience implementing the GCLP Guidelines. The results of the 2017 survey were instrumental in informing changes to GCLP audit activities and promoting harmonization in the approach to laboratory oversight.

A key finding from the 2023 survey results is the preference for hybrid GCLP training, encompassing face-to-face and online modules. Overall, both surveys acknowledged satisfaction with applying and implementing GCLP Guidelines. The need to effectively disseminate information about DAIDS laboratory oversight requirements to support the improved implementation of GCLP Guidelines was notable from both survey results.

The collaborative team of laboratory experts and the integrated oversight approach promote knowledge-sharing and accountability to support the application of the GCLP Guidelines and compliance monitoring. The systematic implementation of the integrated laboratory oversight activities helped identify valuable lessons for improving laboratory performance and opportunities to strengthen quality oversight for laboratories participating in clinical research or trials.

## Introduction

As a sponsor of many clinical investigations, the United States (U.S.) National Institute of Allergy and Infectious Diseases (NIAID) Division of AIDS (DAIDS) aims to ensure the optimal and consistent conduct of clinical research or trials, obtain reliable data critical for the meaningful interpretation of trial findings, and ensure the safety of trial participants and laboratory staff. DAIDS (Sponsor) responsibilities^1^ include promoting the accurate reconstruction of a trial for its submission to a regulatory body such as the Food and Drug Administration, European Medicines Agency (EMA), or South African Health Products Regulatory Authority. To attain these goals, DAIDS developed the Good Clinical Laboratory Practice (GCLP) Guidelines (herein referred to as the “GCLP Guidelines”) to establish a standardized and systematic approach for overseeing laboratories participating in DAIDS-sponsored clinical research or trials.^2–5^

The DAIDS GCLP Guidelines, adapted from the British Association of Research Quality Assurance (BARQA) GCLP guidelines,^3–4^ embrace both the research and clinical laboratory expectations and encompass applicable portions of International Council for Harmonization Good Clinical Practice (GCP), Good Laboratory Practice (GLP), Clinical Laboratory Improvement Amendments (CLIA), and other guidance materials.^2^ GLP standards ensure the consistency and reliability of test data generated by nonclinical laboratories, focusing on the organization and management of laboratory operations, whereas GCP supports quality standards for conducting clinical trials involving human subjects. GCLP, an extension of GLP and GCP, supports the quality management of clinical laboratory testing within the context of clinical research studies. In the United States, CLIA establishes quality standards for clinical laboratories; hence, CLIA-compliant laboratories participating in DAIDS studies are exempted from GCLP compliance.^6–9^ The GCLP Guidelines support a robust quality management system, including external quality assurance (EQA) requirements, assay validation, equipment maintenance, laboratory personnel training and safety, and quality management plan.^5^

To enhance the effective implementation of GCLP Guidelines, the DAIDS Integrated Laboratory Oversight Framework (Framework) was established. The Framework applies a systematic risk-based monitoring (RBM) approach to the oversight of laboratory performance throughout the life of the clinical research or trial. The RBM approach, a proactive strategy to performing onsite and centralized clinical trial monitoring, focuses on identifying risks early in the study and implementing strategies to address them effectively before they become serious.^10^ The approach is dynamic, more readily facilitating continual improvement in trial conduct and oversight.

**FIG. 1.**
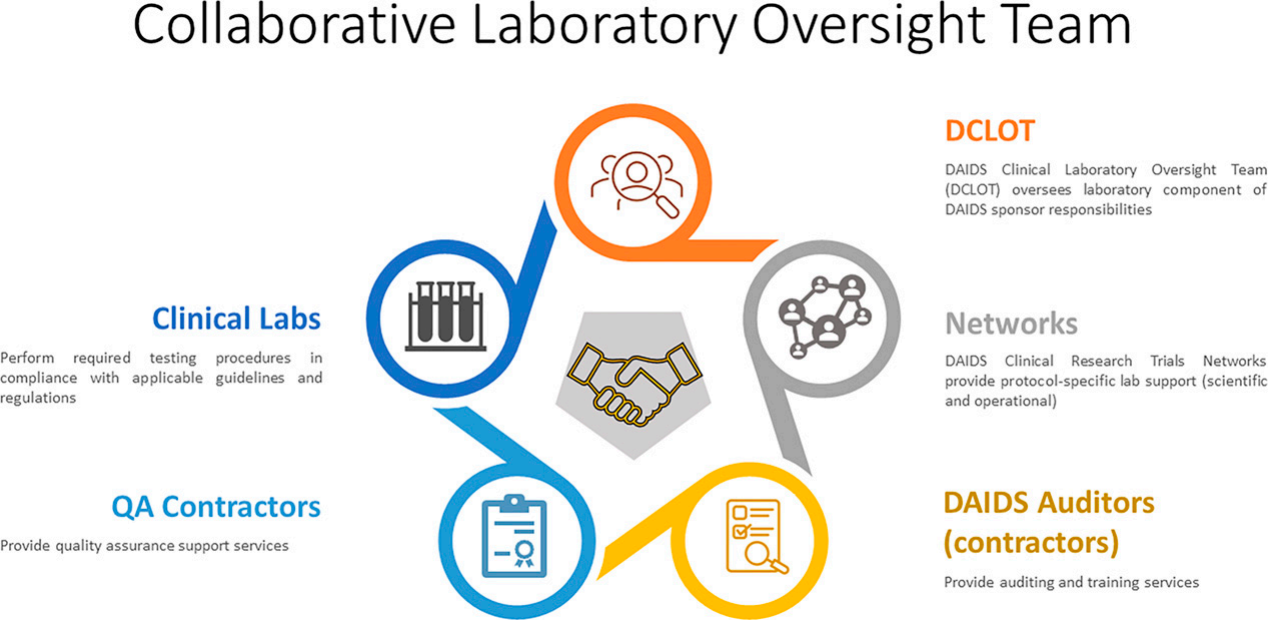
The schematic diagram illustrates the collaborative laboratory oversight team components that provide guidance and assess GCLP compliance of laboratories supporting DAIDS-funded clinical research or trials. GCLP, Good Clinical Laboratory Practice; DAIDS, Division of Aids.

**FIG. 2.**
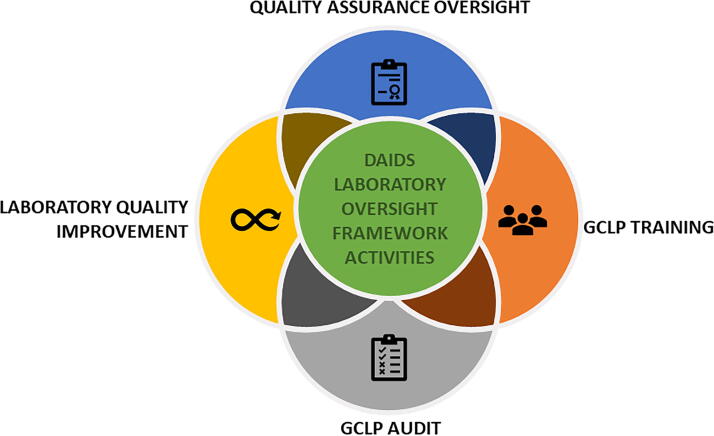
The schematic diagram illustrates the four primary activities of the DAIDS Laboratory Oversight Framework: Quality Assurance Oversight, GCLP Training, GCLP Audit, and Laboratory Quality Improvement.

The collaborative oversight team (oversight team), comprising laboratory experts from the DAIDS Clinical Laboratory Oversight Team (DCLOT), DAIDS Clinical Research Trials Networks (Networks),^11–13^ DAIDS auditors (contractors), QA contractors, and clinical laboratories, supports the implementation and oversight of the robust risk-based Framework (Fig. 1). The Framework includes four activities: *Quality Assurance (QA) Oversight, GCLP Training, GCLP Audit*, and *Laboratory Quality Improvement*^7^ (Fig. 2).

DCLOT, as part of the DAIDS Sponsor responsibility, is required to lead the implementation of the Framework activities, manage the quality of services and communication processes, and ensure compliance with DAIDS clinical laboratory requirements. Fostering effective collaboration among the oversight team (DCLOT and external partners) involves open communication, providing continuous training opportunities to enhance the implementation of GCLP Guidelines, and emphasizing the importance of continuous quality improvement to evaluate implementation processes and identify areas for improvement.

**FIG. 3.**
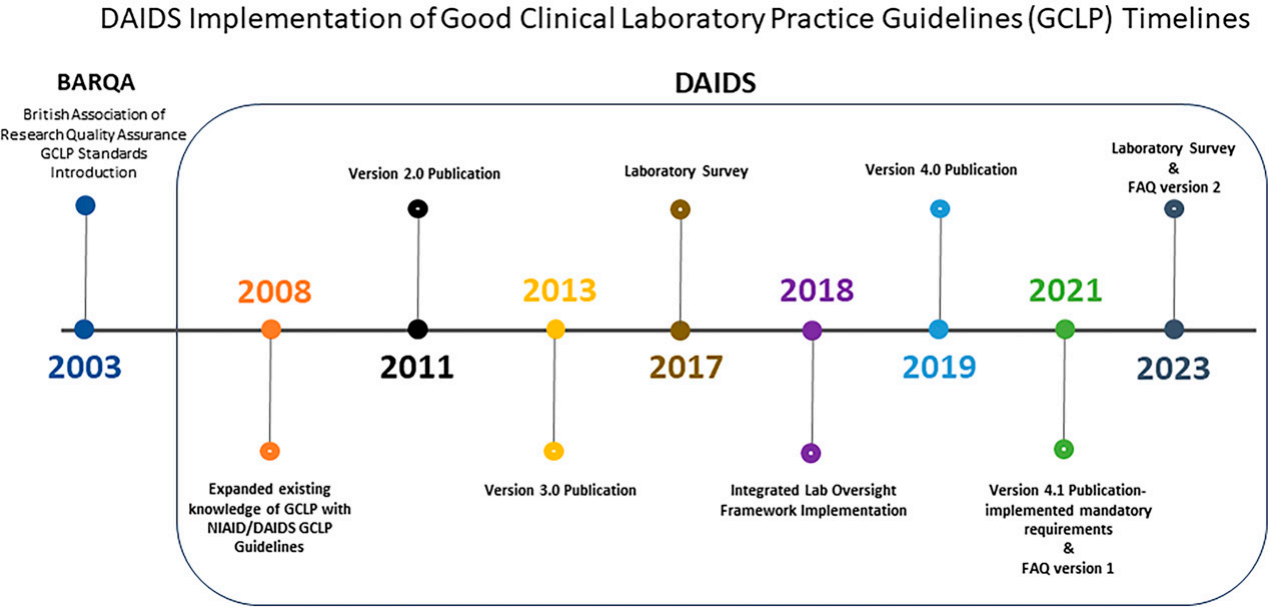
The schematic diagram illustrates the DAIDS history of development, application, and implementation timeline of the GCLP Guidelines from 2003 to date.

This article describes a novel risk-based approach to laboratory oversight using the Framework and the oversight team. The application of the GCLP Guidelines and DCLOT perspective on the collaborative implementation and evaluation efforts, including challenges and opportunities for improvement, are described. The history of development, application, and implementation timeline of the GCLP Guidelines spans over 15 years (Fig. 3). The procedures and processes for implementing the Framework activities in collaboration with external partners are described in a complementary article.^14^

## Application of the DAIDS GCLP Guidelines

The oversight team activities, essential for successfully applying the GCLP Guidelines, include developing harmonized policies and procedures to enhance effective implementation efforts. Laboratories participating in DAIDS-sponsored clinical research or trials, except U.S. laboratories regulated under CLIA, are evaluated for GCLP compliance to ensure satisfactory performance throughout the life of the clinical research or trials.^7–8^ Implementation of GCLP Guidelines and policies promotes the generation of accurate and reliable clinical research data in an environment conducive to study reconstruction.^5,7–9^

The GCLP Guidelines emphasize the development and implementation of standardized procedures for all laboratory processes to ensure consistent and reliable results. These span the spectrum of activities, including establishing and implementing quality control measures to ensure proper specimen management and quality of sample integrity without contamination or degradation. This in turn allows accurate, precise, and reliable test results. Continuous training and competency assessment of laboratory personnel contribute to staff knowledge of GCLP principles and ensure ongoing assessment of performance to identify areas for improvement. Laboratory compliance with GCLP Guidelines demonstrates a commitment to quality, data integrity, reliability, participant and laboratory staff safety, international standards, and regulatory compliance.^5^

Documentation of all procedures and test results ensures data traceability, reproducibility, and accountability throughout the laboratory testing process. The GCLP Guidelines require identifying, assessing, mitigating, and reporting potential risks and hazards associated with laboratory processes to the oversight team to avoid or minimize the adverse impacts on the accuracy and reliability of clinical research or trial results. Laboratory participation in DAIDS-approved EQA programs, such as proficiency testing schemes and interlaboratory comparisons, sets a benchmark for evaluating laboratory performance.^14–15^ The GCLP Guidelines establish minimum expectations for satisfactory laboratory performance and facilitate comparisons across different laboratories.

The laboratories participating in DAIDS-sponsored clinical research or trials may also be required to comply with other applicable local, regional, or international regulations or standards. A comparison of the current version of the DAIDS GCLP Guidelines with GLP, CLIA, College of American Pathologists, International Organization for Standardization (ISO 15189:2012), EMA GCLP, World Health Organization-GCLP (BARQA), and the South African National Accreditation System revealed that DAIDS GCLP Guidelines align with the compared standards.^14^ Compliance with the GCLP Guidelines helps to ensure compliance with U.S. and international laboratory requirements and regulatory frameworks.

## The Risk-Based Oversight Framework Activities

A risk-based approach to monitoring focuses on sponsor oversight activities for preventing or mitigating important and likely risks to data quality and processes critical to human subject protection and trial integrity. The key components of the Framework include QA Oversight, GCLP Training, GCLP Audit, and Lab Quality Improvement.

The *QA Oversight* activities, including EQA program participation, support various steps of study implementation and performance throughout the life of the clinical research or trial. Participation in EQA programs enhances the sponsor’s confidence in the reliability of laboratory test results. Optimal EQA schemes are applied and monitored for clinical research or trial-specific laboratory tests by comparing test performance to a reference or peer group of laboratories. The performance in EQA is likely to reflect on the quality of laboratory testing and help identify any potential issues in the laboratory testing processes. Support is provided to the laboratories to implement Corrective Action and Preventative Action when performance is not optimal. The oversight team provides resources, guidance, and training to support laboratories and promote satisfactory performance in EQA.^14^

*The GCLP Training* includes online training through e-Learning modules and face-to-face (F2F) training. Although face-to-face training offerings are limited and available by request, GCLP e-Learning modules are currently available in English, Spanish, and Portuguese to improve access and comprehension. To date, the numbers of users who completed the English, Spanish, and Portuguese online training are 3927, 221, and 375, respectively. Additional training opportunities may be provided to address DAIDS clinical research or trial-specific requirements and performance-based challenges.^14^

The risk-based *GCLP Audit* objectively assesses whether a laboratory is operating in compliance with regulatory requirements, accreditation standards, and GCLP Guidelines. The audit also helps to determine the readiness and ongoing participation of laboratories in DAIDS-sponsored clinical research or trials. Audits can be external (onsite or remote), conducted by DAIDS audit contractors or oversight team representatives, or internal (self-assessment). During an external audit, the laboratory is evaluated for ongoing performance, investigated for the root cause analysis of laboratory-related events (e.g., for-cause audit), or assessed and trained in preparation for a regulatory inspection. DCLOT, with support from relevant oversight team members, identifies and assists the laboratories in resolving audit findings.

The oversight team, led by DCLOT, monitors the *Laboratory Quality Improvement* and laboratory performance using EQA, audits, and training compliance throughout the life of the clinical research or trial.^14^ The quality improvement activities focus not merely on historical data but also on potential future risks, thus offering a more strategic, efficient, and value-adding approach to regulatory compliance. In addition, DCLOT members, as representatives from DAIDS, the sponsor of the clinical research or trial, occasionally attend the GCLP audit as observers to assess the overall conduct of the audit, observe the interaction between the auditor and laboratory staff, and evaluate the effectiveness of the implementation of GCLP Guidelines. Also, self-administered online surveys are conducted (as needed) to evaluate the effectiveness of the oversight team’s implementation efforts and identify any areas that require improvement. The surveys offer an opportunity to incorporate the laboratory staff’s perspective on the value of the integrated laboratory oversight activities and help identify gaps and deficiencies to inform continuous quality improvement. Together, these activities promote interactions with a wide range of stakeholders, leading to more open lines of communication, improving relationships, and fostering a collaborative environment.

## Implementation and Evaluation

The 2017 and 2023 surveys assessed laboratory staff awareness, knowledge, and understanding of the GCLP Guidelines. The survey, developed by DCLOT with support from the oversight team, used qualitative, quantitative, and closed- and open-ended questions to seek information about laboratory staff’s experience with GCLP audits, training, and quality assurance processes. The overarching goal of surveys was to improve the quality and effectiveness of laboratory oversight activities. Laboratory staff at DAIDS-sponsored clinical research sites completed the questionnaire using Survey Monkey.^16^ The survey was sent to over 175 laboratories participating in DAIDS-sponsored clinical research or trials. The response rate was 87% (102/117) and 97% (108/111) for the 2017 and 2023 surveys, respectively. Several laboratories participated in more than one of the major DAIDS clinical trials networks—Advancing Clinical Therapeutics Globally for HIV/AIDS and Other Infections, HIV Prevention Trials Network, HIV Vaccine Trials Network, and International Maternal Pediatric Adolescent AIDS Clinical Trials.^11–12^

**FIG. 4.**
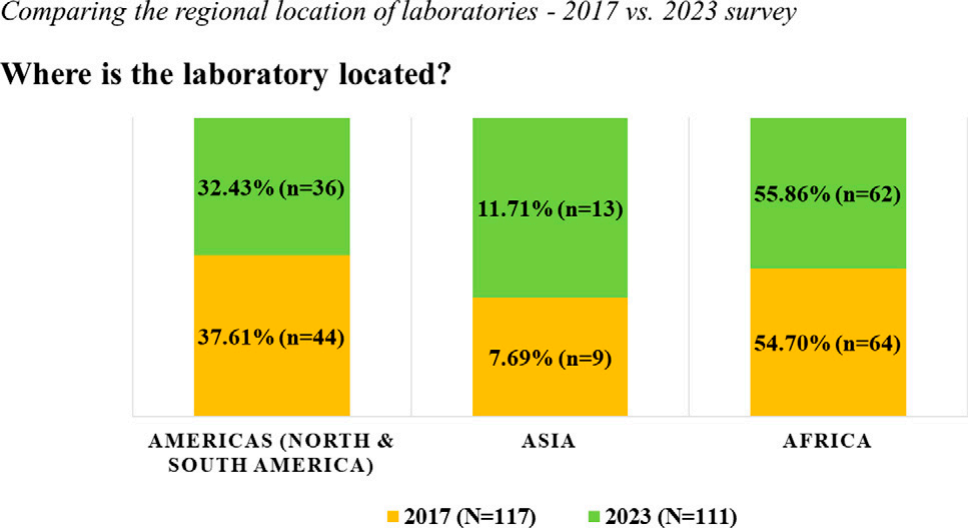
The schematic diagram illustrates the laboratory location on continent-level data from two self-administered online surveys conducted in 2017 (*orange*) and 2023 (*green*) to assess the laboratory staff’s experience implementing the GCLP Guidelines.

**FIG. 5.**
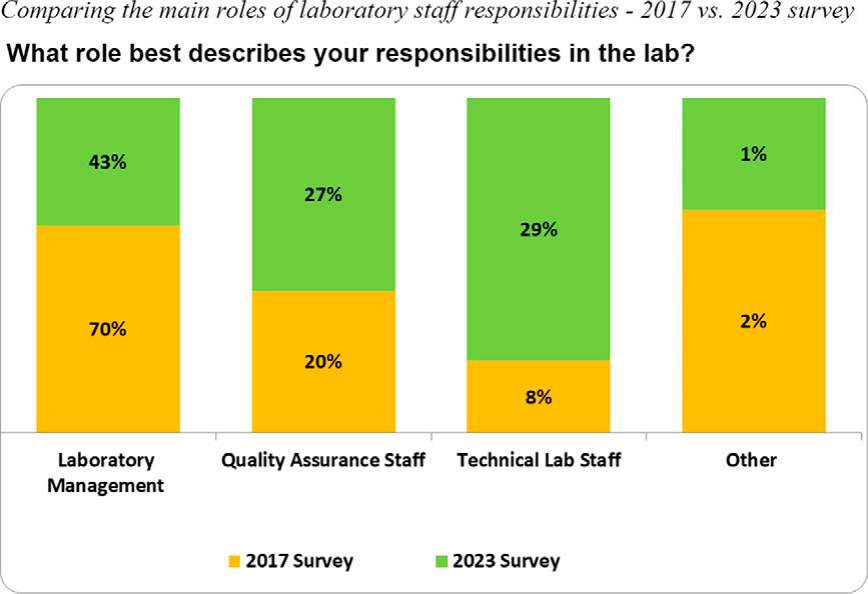
The schematic diagram illustrates the four main roles to describe laboratory staff responsibilities: lab management, quality assurance staff, technical laboratory staff, and other (any nonlaboratory role) from two self-administered online surveys conducted in 2017 (*orange*) and 2023 (*green*).

Contrary to the anonymous approach utilized in 2017, the 2023 survey used a Harmonized Identification (HID), a system to identify and harmonize laboratory names. The four-digit HID number was used to support targeted follow-up assistance to laboratories. The geographic distribution of participating laboratories across both surveys included those in Africa (55%), Asia (8%–12%), and the Americas (32%–38%) (Fig. 4). Due to DAIDS site expansion efforts, the number of laboratories in Asia increased by 4% from 2017 to 2023, compared to a 5% decrease in the Americas, and remained relatively unchanged in the African region. For both surveys, more than 50% of the laboratories were certified by at least one local or international regulatory/accreditation body. The survey respondents represented laboratory management staff (director, manager, or head of facility), QA staff, technical laboratory staff, and other (any nonlaboratory role), as shown in Figure 5. Laboratory management staff provided 70% of the survey responses in 2017, which decreased to 43% in the 2023 survey, coinciding with a notable 21% increase in responses from technical laboratory staff.

Overall, results were comparable between surveys. Approximately 96% of respondents agreed that the DAIDS laboratory audit findings were of value in improving performance, noting that the audit process was clear and well-defined, and the frequency of audits was adequate. The responses also show that the laboratory staff were knowledgeable of the GCLP Guidelines and the significance of the online training modules in effectively increasing their understanding and compliance. The survey respondents indicated a preference for F2F GCLP training (∼55%) versus online training (∼28%) in 2017, and for hybrid (online and F2F) training (∼37%) versus F2F (∼ 35%) versus online (∼27%) in 2023. The reasons cited for the hybrid training included the increased flexibility, ease of access, and convenience of self-paced online learning and the occasional F2F training opportunities to help foster collaboration and relationship-building among peers.

Implementation of the recommended actions from the 2017 survey results included the need to streamline the GCLP audit process and harmonize the GCLP Guidelines with other regulatory standards and guidelines. The 2023 survey evaluated the actions implemented from the 2017 survey. Both surveys revealed a positive trend in the awareness and knowledge of GCLP Guidelines and supporting processes. However, gaps in the dissemination of information were identified, indicating the need for improved communication procedures. For instance, although the *DAIDS GCLP Training-related Frequently Asked Questions (FAQs)*^17^ was released to all participating laboratories using established DAIDS communication resources, only 84% of respondents from the 2023 survey indicated awareness of the guidance. Moving forward, the timely dissemination of new guidelines and requirements is key to promoting adherence to GCLP Guidelines. Improved communication channels and resources that embrace technology and emphasize continual learning and training underscore the value of standardization and endorse proactive communication to produce high-quality, meaningful scientific research responsible for maintaining public trust.

## Implementation Challenges and Opportunities for Improvement

Despite global challenges experienced during the COVID-19 pandemic,^18^ laboratories were expected to comply with GCLP Guidelines and implementation activities. The increased testing demand during the peak of COVID-19 infections strained laboratory resources and personnel. The application of the risk-based DAIDS Laboratory Oversight Framework activities helped avert disruptions in clinical laboratory testing in DAIDS-sponsored clinical research or trials during the COVID-19 pandemic and ensured continuous participation and satisfactory laboratory performance. Several challenges were noted, including delayed GCLP audits, EQA testing disruptions, and reagent shortages due to supply chain issues.

Depending on a global network of suppliers, the clinical laboratory’s supply chain was vulnerable to disruptions due to natural disasters, shortages, or geopolitical conflicts.^19^ Laboratories are more likely to continue operating during unexpected supply contingencies when laboratory management responds swiftly and effectively to such disruptions. Although predicting global and local supply chain vulnerabilities can be challenging, developing a contingency plan can ensure that laboratory testing continues uninterrupted. The risk-based oversight framework established mitigation strategies for laboratories participating in DAIDS-sponsored clinical research or trials. Established laboratory backup plans helped identify products or engage laboratories that could support timely testing activities with minimal disruptions. Moreover, enhanced information sharing among stakeholders using the collaborative oversight team helped to avoid the same issue from occurring at different laboratories.

Ongoing laboratory oversight during the recent COVID-19 pandemic required remote GCLP audits to allow the sponsor to meet oversight requirements. Implementing remote audits to mitigate travel restrictions and social distancing guidelines during the pandemic helped overcome the challenges of in-person (onsite) audits and supported the review of documents and assessing compliance with DAIDS GCLP Guidelines without the need for physical presence at the audited laboratory. However, remote audits limit direct observation of GCLP Guidelines expectations (such as observation of testing procedures and complete review of physical facilities) compared to onsite audits. Also, the requirement to scan and upload documents for auditor review during remote audits can be challenging for some laboratories. While the remote audit complemented onsite audit requirements during the pandemic, the process provides an additional tool for monitoring and assessing laboratory performance.

Strengthening the *Laboratory Quality Improvement* component of the oversight framework provides a robust and objective approach to monitoring the oversight team activities^14^ and laboratory performance. A recent update to the D*AIDS GCLP-related Training FAQs*^17^ clarified mandatory GCLP training requirements and provided guidance to U.S. and non-U.S. laboratory staff performing testing and processing for DAIDS-sponsored clinical research or trials. Continuous training and the availability of online training modules and GCLP Guidelines in additional languages will enhance awareness and knowledge of DAIDS GCLP Guidelines and improve laboratory performance. Evaluating the risk-based oversight framework requires monitoring key performance indicators (KPIs) to objectively assess laboratory performance overtime.^20^ The oversight team will develop key quality indicators to measure ongoing performance across all framework activities as part of a comprehensive quality management system.^21^ The KPIs will be tracked regularly, reviewed, and analyzed to help identify areas of improvement.

Change is inevitable in science, whether there is a new policy for the staff, the implementation of new systems, or new regulations that affect clinical research. The GCLP Guidelines are continuously updated accordingly to communicate policies and procedures that are clear, concise, and easy to understand. Sponsors need to articulate why the change is necessary, precisely what is changing, what is staying the same, and what steps staff need to take to comply with the new requirements. In addition, there is a need for the sponsors to state the consequences of noncompliance if revised policies are to be followed closely.

## Conclusion

The application of GCLP Guidelines and the risk-based oversight framework, along with the collaborative team efforts, can strengthen laboratory performance and ensure the reliability, accuracy, and consistency of clinical data. The framework also establishes a systematic approach to assessing laboratory readiness, monitoring and evaluating ongoing laboratory performance, and enhancing feedback mechanisms to strengthen quality control measures. The lessons learned from the implementation of the GCLP Guidelines described in the complementary article^14^ informed opportunities to address challenges and helped strengthen laboratory performance. The oversight team’s efforts foster a culture of teamwork, open communication, sharing of diverse expertise and perspectives, innovative problem-solving, improved efficiency, enhanced quality, and oversight of DAIDS-sponsored clinical research or trials.

The GCLP Guidelines have helped build a strong foundation for implementing quality management systems and successful laboratory performance, and guide capacity building of the laboratories.^22^ The process has ensured that laboratory testing is conducted successfully, generating high-quality research data. The structured approach to laboratory qualification and monitoring provides ongoing performance assessment, and ensures accurate, precise, reproducible data to guarantee sponsor confidence and adherence to regulatory agency requirements. Lessons learned from the laboratory oversight approach can serve as a blueprint for other organizations to develop and implement quality oversight across multiple laboratories.
